# Comparison of LDPI to SPECT perfusion imaging using ^99m^Tc-sestamibi and ^99m^Tc-pyrophosphate in a murine ischemic hind limb model of neovascularization

**DOI:** 10.1186/s13550-016-0199-2

**Published:** 2016-05-27

**Authors:** Geert Hendrikx, Mark H. Vries, Matthias Bauwens, Marijke De Saint-Hubert, Allard Wagenaar, Joël Guillaume, Levinia Boonen, Mark J. Post, Felix M. Mottaghy

**Affiliations:** Department of Nuclear Medicine, Maastricht University Medical Centre (MUMC+), Postbox 5800, AZ 6202 Maastricht, The Netherlands; Department of Physiology, CARIM, Maastricht University, Maastricht, The Netherlands; Cardiovascular Research Institute Maastricht (CARIM), Maastricht University, Maastricht, The Netherlands; Department of Nuclear Medicine, University hospital, RWTH University, Aachen, Germany

**Keywords:** Laser Doppler perfusion imaging (LDPI), Single-photon emission computed tomography (SPECT), Hind limb ischemia, Perfusion recovery, Overestimation of therapeutic window

## Abstract

**Background:**

We aimed to determine the accuracy of laser Doppler perfusion imaging (LDPI) in an animal model for hind limb ischemia.

**Methods:**

We used a murine (C57Bl/6 mice) ischemic hind limb model in which we compared LDPI with the clinically used ^99m^Tc-sestamibi SPECT perfusion imaging (*n* = 7). In addition, we used the SPECT tracer ^99m^Tc-pyrophosphate (^99m^Tc-PyP) to image muscular damage (*n* = 6).

**Results:**

LDPI indicated a quick and prominent decrease in perfusion immediately after ligation, subsequently recovering to 21.9 and 25.2 % 14 days later in the ^99m^Tc-sestamibi and ^99m^Tc-PyP group, respectively. ^99m^Tc-sestamibi SPECT scans also showed a quick decrease in perfusion. However, nearly full recovery was reached 7 days post ligation. Muscular damage, indicated by the uptake of ^99m^Tc-PyP, was highest at day 3 and recovered to baseline levels at day 14 post ligation. Postmortem histology supported these findings, as a significantly increased collateral diameter was found 7 and 14 days after ligation and peak macrophage infiltration and TUNEL positivity was found on day 3 after ligation.

**Conclusions:**

Here, we indicate that LDPI strongly underestimates perfusion recovery in a hind limb model for profound ischemia.

## Background

Numerous ischemic hind limb models have been developed in mice [1–10], rats [11, 12], and rabbits [1] in order to study post-ligation perfusion recovery through neovascularization. Most hind limb ischemia studies have been conducted in mice and rats and regardless of the studied mode of neovascularization (i.e., arteriogenesis or angiogenesis), and follow-up of post-ligation perfusion recovery is often performed by laser Doppler perfusion imaging (LDPI) [2–6, 9, 10, 13, 14]. Other methods frequently used in pre-clinical research for the assessment of post-ligation perfusion recovery are angiography [3, 4, 9–11, 14, 15], microsphere injections [2, 4, 11, 12, 15], contrast-enhanced ultrasound [16], micro CT [10], and magnetic resonance imaging (MRI) [14]. However, despite the variety in methods to monitor perfusion recovery in the ischemic hind limb, LDPI remains the most frequently used. The main reasons are that LDPI is a fast, efficient, and non-invasive technique to document extremity blood-flow [17]. However, since LDPI is limited by the penetration depth of the laser light [8, 18], allowing measurement of superficial skin perfusion only, the question arises whether LDPI scanning is accurately reflecting the incremental perfusion improvement of skeletal muscles through arteriogenesis in the upper hind limb or angiogenesis in the lower hind limb of the animal.

Positron emission tomography (PET) and single-photon emission computed tomography (SPECT) are non-invasive and radioisotope-based nuclear imaging techniques that can be used to image changes in tissue blood flow, which can provide early, sensitive, and specific detection of ischemic diseases at the molecular level [19, 20]. With increasing availability of better and dedicated small animal cameras as well as the development of new radiotracers, these nuclear imaging techniques provide excellent tools for perfusion imaging.

Here, we used the SPECT perfusion tracer technetium-99m sestamibi (^99m^Tc-sestamibi) to study whether LDPI accurately reflects perfusion recovery in a murine ischemic hind limb model for arteriogenesis. Additionally, we used technetium-99m pyrophosphate (^99m^Tc-PyP) as a molecular tracer for muscular damage as it binds to hydroxyapatite crystals in damaged myocytes [21, 22]. Acquired 3-dimensional (3D) information on perfusion and myocyte damage provided by SPECT scans may prove to be a useful addition to standardized pre-clinical perfusion recovery analysis by LDPI.

## Methods

### Animal model

C57Bl/6 mice were anesthetized with isoflurane (1.5–2 %) throughout the whole surgical procedure to induce hind limb ischemia. Analgesia was applied 30 min before and 6 h after the procedure with Temgesic (0.5 mg/BW). In short, a longitudinal incision was made in the skin overlying the middle portion of the right hind limb of the mice. The femoral artery was dissected for several millimeters in length from the femoral nerve and femoral vein. The artery was ligated proximal to the superficial epigastric artery by electrocoagulation. Subsequently, the skin was closed with a continued suture. Next, a longitudinal incision was made in the skin just above the knee of the right hind limb. The femoral artery was dissected for several millimeters in length from the femoral nerve and femoral vein. The artery was ligated distal from the bifurcation of the saphenous artery and the popliteal artery by electrocoagulation. Seven C57Bl/6 mice were used in the ^99m^Tc-sestamibi group, and six C57Bl/6 mice were used in the ^99m^Tc-PyP group.

Animals were held under the guidelines of the animal care facility (Maastricht University) with unlimited access to food and drinking water. All animal experiments were approved by the Committee for Animal Welfare of the Maastricht University conform the Directive 2010/63/EU of the European Parliament.

### Laser Doppler perfusion imaging

**Fig. 1:**
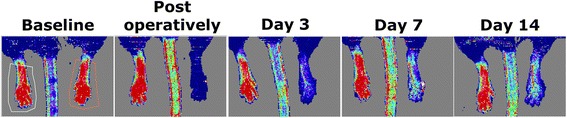
A typical representation of perfusion recovery in the mouse hind limb over time as can be seen by LDPI. A representative ROI placement for LDPI is shown in the baseline panel

Under standardized conditions, blood flow in the hind limbs of all animals was measured using LDPI (Moor Instruments, UK). Measurements were performed 4 days prior to the surgical procedure (baseline scan), directly after the surgical procedure and at post-operative days 3, 7, and 14. During the measurements, mice were under anesthesia (isoflurane: induction 2.5 %; maintenance 1.5 %) and placed on a heating pad in a climate controlled chamber (37 °C) to ensure minimal variation between flow measurements. The perfusion ratio per time point was calculated by dividing the perfusion of the ligated (ischemic) by the non-ligated (non-ischemic) hind limb. Regions of interest (ROIs) were drawn manually around the foot of the ischemic hind limb and the contralateral non-ischemic foot (Fig. 1 baseline panel).

### SPECT tracers

#### ^99m^Tc-sestamibi

^99m^Tc-sestamibi was prepared from a lyophilized sestamibi kit according to manufacturer guidelines (Mallinckrodt Medical, Petten, The Netherlands). Quality controls were satisfactory for every synthesis (radiochemical purity >98 %). The activity was 59.8 ± 21.0 MBq (mean ± SD).

#### ^99m^Tc-pyrophosphate

Six milliliters of 0.9 % NaCl solution was added to the lyophilized PyP-kit (Technescan PYP, Mallinckrodt Medical, Petten, The Netherlands). One hundred fifty microliters of the stannous ion containing PyP kit (10 times diluted) was injected through a custom-made tail vein catheter in order to reduce red blood cell-bound proteins. Fifteen minutes post-stannous PyP injection, freshly eluted pertechnetate (activity 55.5 ± 19.4 MBq, mean ± SD) was injected through the same tail vein catheter. In vivo formed ^99m^Tc-stannous PyP binds to hydroxyapatite crystals in damaged cells, thereby acting as a molecular marker for cellular damage.

### SPECT imaging

#### Imaging protocols

Mice were anesthetized with isoflurane (induction 2.5 %; maintenance; 1.5 %), and a catheter was placed in the tail vein and the animals were positioned in the micro-SPECT camera (MiLabs, Utrecht, The Netherlands). Imaging of the mice in the ^99m^Tc-sestamibi group and the ^99m^Tc-pyrophosphate group was performed in 1 time frame of 30 min.

#### SPECT reconstruction

U-SPECT-II reconstruction software version 2.38 (MiLabs, Utrecht, The Netherlands) was used to reconstruct images [23]. ^99m^Tc images were reconstructed by selecting the photopeak (PP) and background (BG) windows. We used a PP window of 126–154 KeV and BG windows of 115–120 and 190–200 KeV.

#### SPECT quantification

To allow quantification of radiotracer uptake in vivo, conversion factors (CFs) were determined for the 1.0 mm collimator system in a representative phantom (20 ml) for ^99m^Tc. During the SPECT reconstruction, the total amount of counts was distributed over the different voxels, resulting in a counts/voxel expression for the average concentration of counts. Taking into account, the voxel volume (Vv) and the CFs are calculated using Eq. (1):

1 $$ \mathrm{Conversion}\ \mathrm{Factor}\ \left(\mathrm{MBq}\right){\scriptscriptstyle \frac{\mathrm{Activity}\ \mathrm{concentration}\ \left(\frac{\mathrm{MBq}}{\mathrm{ml}}\right)}{\frac{\mathrm{SPECT}\ \mathrm{counts}\ \mathrm{per}\ \mathrm{voxel}}{\mathrm{Vv}\ \left(\mathrm{ml}\right)}}} $$

Using this formula, we were able to determine the following CF: CF_Tc_ = 640 MBq.

Using the PMOD 2.95 view tool (PMOD Technologies, Zürich, Switzerland), volumes of interest (VOIs) were drawn in the lower, upper part of the hind limb (i.e., the area of collateralization). Each VOI had a volume of 27 mm^3^. For both the ^99m^Tc-sestamibi and ^99m^Tc-PyP images, the uptake was measured for each VOI and expressed as mean standardized uptake value (SUV_mean_) using Eq. (2).

2 $$ \mathrm{S}\mathrm{U}\mathrm{V}=\left[\mathrm{SPECT}\ \mathrm{counts}\ \mathrm{per}\ \mathrm{ml} \times \mathrm{C}\mathrm{F}\right]\left(\mathrm{MBq}/\mathrm{ml}\right)\times \left[\frac{\mathrm{Body}\ \mathrm{weight}\ \left(\mathrm{g}\right)}{\mathrm{Injected}\ \mathrm{dose}\ \left(\mathrm{MBq}\right)}\right] $$

For body weight, we assumed that 1 g of body weight equalled 1 ml.

### Histology

A group of 11 C57Bl/6 mice was used solely for histological purposes at follow-up of 0, 3, 7, or 14 days with *n* = 2, 3, 3, and 3, respectively. After euthanasia, tissues were harvested and prepared for histological examination of paraffin sections.

#### α-Smooth muscle actin (αSMA)

Tissue sections (5 μm) were routinely stained for αSMA and CD68. In short, tissue sections for the αSMA staining were deparaffinized in xylene, rehydrated in ethanol, and rinsed in distilled water. Endogenous peroxidases were blocked in 0.3 % hydrogen peroxide in PBS. Subsequently, slides were incubated overnight with the 1A4 anti-smooth muscle actin antibody (Sigma-Aldrich, Zwijndrecht, The Netherlands) in a dilution of 1:3000. On the following day, the slides were incubated with the peroxidase-conjugated rat anti-mouse secondary antibody (dilution 1:250), and sites of SMA expression were visualized by chromogenic detection via 3,3′-diaminobenzidine (DAB) (DAKO, Heverlee, Belgium) in a dilution of 1:50. Finally, after dehydration, slides were mounted using Entellan mounting medium (Merck, Darmstadt, Germany). Photomicrographs were acquired using StereoInvestigator software (MBF Bioscience, Williston, USA) on a BX51WI spinning disk confocal fluorescence microscope (Olympus, Tokyo, Japan) with a QIcam color camera (QImaging, Surrey, Canada). In every tissue specimen, the diameter of five collateral arteries was determined using ImageJ (NIH).

#### CD68

After rehydration, transversal tissue sections were blocked in 0.3 % hydrogen peroxide in PBS and 2 % bovine serum albumin in PBS/0.05 % Tween. Non-specific binding of the avidin/biotin system was prevented using the avidin/biotin blocking kit (Vector Laboratories, Burlingame, USA). Subsequently, slides were incubated overnight with the anti-CD68 antibody (Bio-Connect, Huissen, The Netherlands) in a 1:400 dilution. The following day, in successive order, slides were incubated with secondary antibody (1:200 dilution), the avidin/biotin complex (Vectastain ABC kit, Vector Laboratories, Burlingame, USA) (dilution 1:100) and DAB (dilution 1:50) for visualization. Slides were mounted using Entellan mounting medium (Merck, Darmstadt, Germany). Photomicrographs were acquired using StereoInvestigator software (MBF Bioscience, Williston, USA) on a BX51WI spinning disk confocal fluorescence microscope (Olympus, Tokyo, Japan) with a QIcam color camera (QImaging, Surrey, Canada). Counting CD68 positive cells in the peri-muscular fascia was performed using ImageJ (NIH).

#### Terminal deoxynucleotidyl transferase dUTP nick end labeling (TUNEL)

After rehydration, transverse tissue sections were incubated with proteinase K (20 μg/ml in PBS). After washing in PBS, tissue sections were blocked in 20 % fetal bovine serum/3 % bovine serum albumin in PBS. Double- and single-stranded DNA breaks were subsequently enzymatically labeled with fluorescein-dUTP using the in situ cell death detection kit (Roche, Mannheim, Germany) according to manufacturer guidelines. After washing in PBS, the slides were mounted using glycerol mounting medium with 4′,6-diamidino-2-phenylindole (DAPI) and 1,4-diazabicyclo 2,2,2 octane (DABCO) (Abcam, Cambridge, UK). Photomicrographs were acquired using Leica application suite X software (Leica microsystems, Eindhoven, The Netherlands) on a microscope coupled to a computerized morphometry system (Quantimed 570, Leica, Eindhoven, The Netherlands). Counting TUNEL positive cells in the peri-muscular fascia was performed using ImageJ (NIH).

### Statistics

Data were expressed as averages for each group ± standard error of the mean (SEM). Student’s *T* test was performed to test for the significance between ischemic and non-ischemic control data. A two-way analysis of variance (ANOVA) with a Bonferroni post-test was used to test for significant differences in collateral artery size, CD68 positivity, and TUNEL positivity in the adductor muscles between ischemic and non-ischemic limbs. *p* < 0.05 was considered statistically significant. Data were analyzed in GraphPad (GraphPad software, La Jolla, USA).

## Results

### LDPI post femoral artery ligation

Figure 1 illustrates the typical representation of perfusion recovery as seen on LDPI. Following femoral artery ligation, average perfusion ratios immediately dropped to ~2 % of average baseline ratios. Subsequent perfusion recovery as measured over time by LDPI did not differ between the two groups (Fig. 3a and Table 1).

**Table 1:** Overview of the average LDPI ratios ischemic/non-ischemic limb in the ^99m^Tc-sestamibi and ^99m^Tc-PyP scanned group per time point

LDPI			
^99m^Tc-sestamibi group			
Time point	Ratio ± SEM	*p* value	*N*
Baseline	0.98 ± 0.039	NS	7
Post-operatively	0.02 ± 0.004	*p* < 0.001	7
Day 3	0.10 ± 0.023	*p* < 0.001	7
Day 7	0.19 ± 0.035	*p* < 0.001	7
Day 14	0.22 ± 0.016	*p* < 0.001	7
^99m^Tc-PyP group			
Time point	Ratio ± SEM	*p* value	*N*
Baseline	1.00 ± 0.015	NS	6
Post-operatively	0.02 ± 0.002	*p* < 0.001	6
Day 3	0.10 ± 0.006	*p* < 0.001	6
Day 7	0.19 ± 0.008	*p* < 0.001	5
Day 14	0.25 ± 0.022	*p* < 0.001	5

Represented *p* values indicate the difference compared to the non-ischemic limb

*NS* non-significant, *N* the number of animals available for analysis

### SPECT imaging post femoral artery ligation

**Fig. 2:**
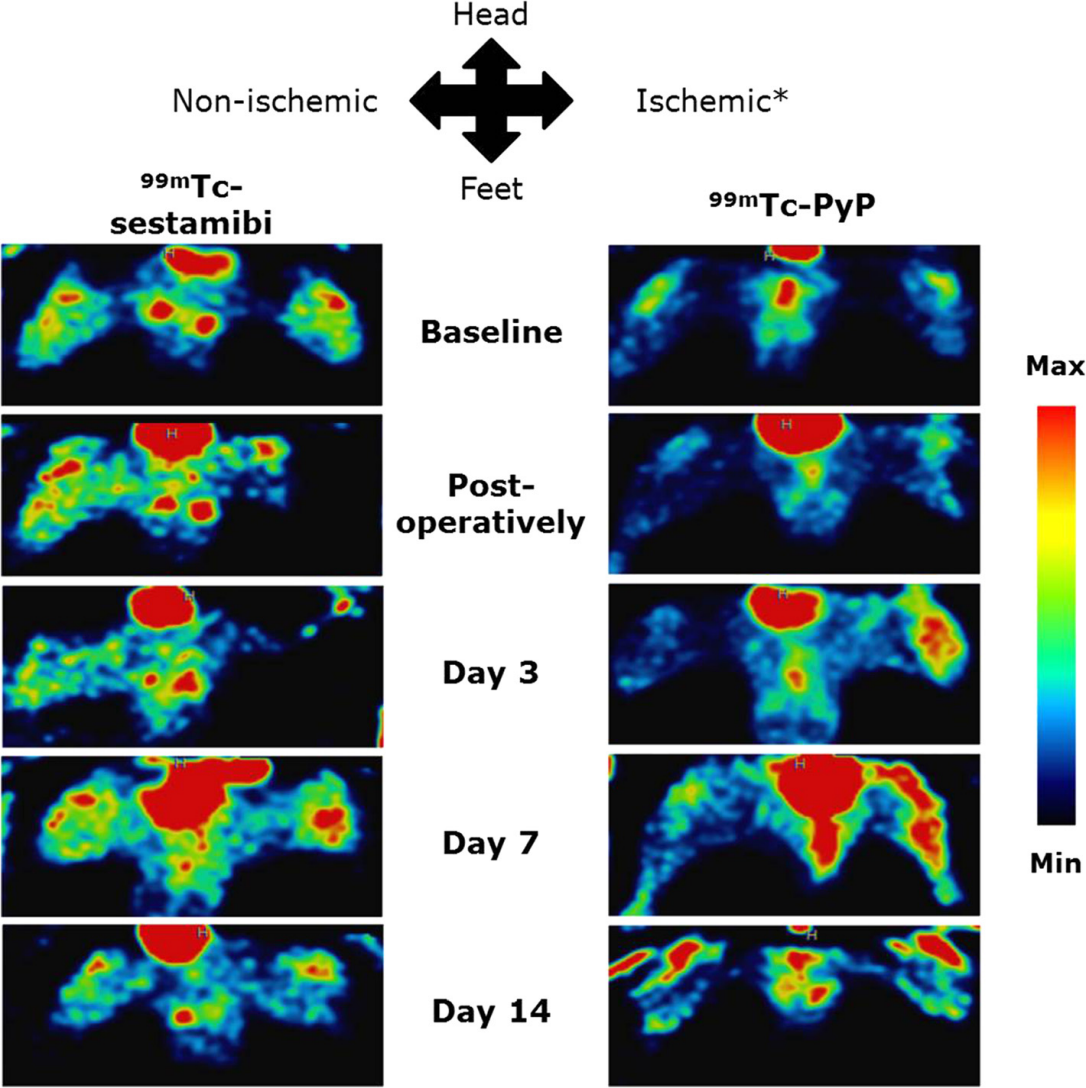
A typical representation of perfusion recovery as seen by ^99m^Tc-sestamibi SPECT and muscular damage as seen by ^99m^Tc-PyP SPECT. In all images, the lower part of the body is displayed in supine position. Image signal intensity is color-coded and relative to the injected dose per animal. *Asterisk* marks the ligated hind limb.

**Fig. 3:**
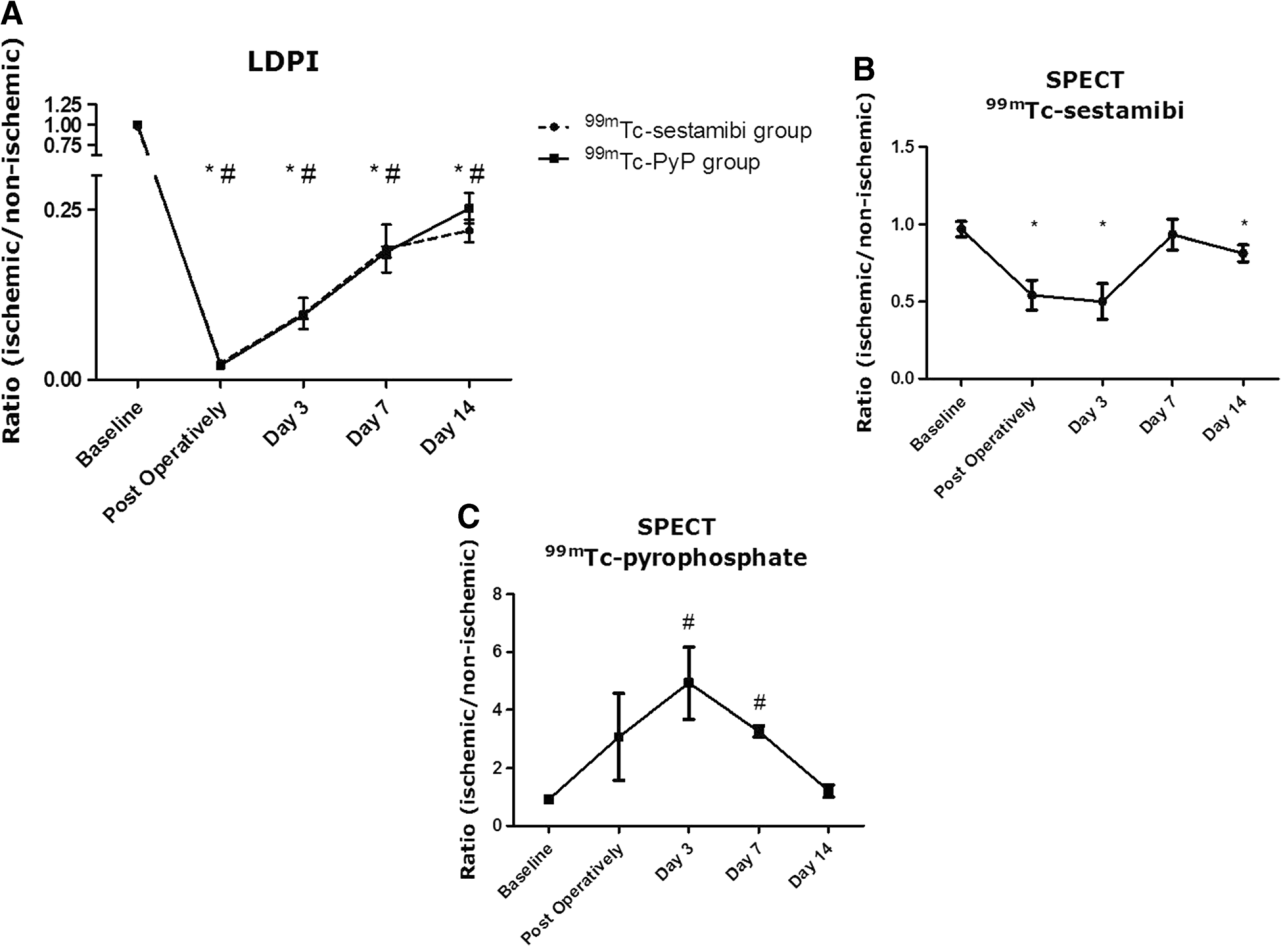
Average LDPI ratios (ischemic/non-ischemic) of both groups, *n* = 7 in the ^99m^Tc-sestamibi group, *n* ≥ 5 in the ^99m^Tc-PyP group (**a**). Average ratios determined from SUVs calculated in the ^99m^Tc-sestamibi scanned group with *n* ≥ 6 for every time point (**b**). Average ratios determined from SUVs calculated in the ^99m^Tc-PyP scanned group with *n* ≥ 3 for every time point (**c**). All data are represented as mean ± SEM. *Asterisk* indicates significantly different from non-ischemic in the ^99m^Tc-sestamibi group while *number sign* is used for significantly different from non-ischemic in the ^99m^Tc-PyP group

To study the perfusion recovery and (clearance of) muscular damage in the total volume of the ischemic versus the contralateral non-ischemic hind limb, we performed quantitative SPECT scans on both groups of mice. In Fig. 2, the perfusion recovery and muscular damage as seen on SPECT images is illustrated. In the ^99m^Tc-sestamibi scanned group, average SUVs in the ischemic hind limb dropped significantly compared to the contralateral non-ischemic hind limb directly after ligation (ratio 0.54 with *p* = 0.007) and remained decreased 3 days post ligation (ratio 0.50 with *p* = 0.006) (Table 2 and Fig. 3b). Remarkably, 7 and 14 days post ligation surgery average SUVs indicated nearly full perfusion recovery (Table 2 and Fig. 3b).

**Table 2:** Overview of the average SUVs (±SEM) in the non-ischemic and ischemic hind limb and the average ratio (±SEM) calculated from SPECT scans in the ^99m^Tc-sestamibi and ^99m^Tc-PyP group per time point

SPECT					
^99m^Tc-sestamibi group					
Time point	SUV non-ischemic	SUV ischemic ± SEM	Ratio ± SEM	*p* value	*N*
Baseline	0.30 ± 0.04	0.29 ± 0.04	0.97 ± 0.05	NS	7
Post-operatively	0.27 ± 0.04	0.16 ± 0.04	0.54 ± 0.10	*p* = 0.007	7
Day 3	0.24 ± 0.04	0.13 ± 0.04	0.50 ± 0.11	*p* = 0.006	6
Day 7	0.30 ± 0.04	0.28 ± 0.04	0.93 ± 0.10	NS	7
Day 14	0.26 ± 0.04	0.21 ± 0.04	0.81 ± 0.05	*p* = 0.022	7
^99m^Tc-PyP group					
Time point	SUV non-ischemic	SUV ischemic	Ratio	*p* value	*N*
Baseline	0.17 ± 0.03	0.15 ± 0.03	0.92 ± 0.04	NS	6
Post-operatively	0.15 ± 0.04	0.35 ± 0.17	3.08 ± 1.50	NS	6
Day 3	0.16 ± 0.04	0.57 ± 0.08	4.93 ± 1.25	*p* = 0.004	5
Day 7	0.13 ± 0.04	0.42 ± 0.10	3.27 ± 0.19	*p* = 0.013	5
Day 14	0.16 ± 0.07	0.18 ± 0.06	1.21 ± 0.21	NS	3

Represented *p* values indicate the difference compared to the non-ischemic limb

*NS* non-significant, *N* the number of animals available for analysis

Average SUVs obtained from ^99m^Tc-PyP SPECT scans showed a different course, probably reflecting uptake of the tracer by damaged skeletal muscle cells. The uptake of ^99m^Tc-PyP in the ischemic limb increased drastically upon ligation and peaked 3 days post ligation surgery (ratio 4.93 with *p* = 0.004) (Table 2 and Fig. 3c). At day 7 post ligation, average SUVs in the ischemic hind limb strongly decreased (ratio 3.27 with *p* = 0.013). The average ratio continued to decrease and reached a value comparable to baseline 14 days post ligation (Table 2 and Fig. 3c).

### Postmortem analysis of collateral artery size, macrophage infiltration, and DNA fragmentation

**Fig. 4:**
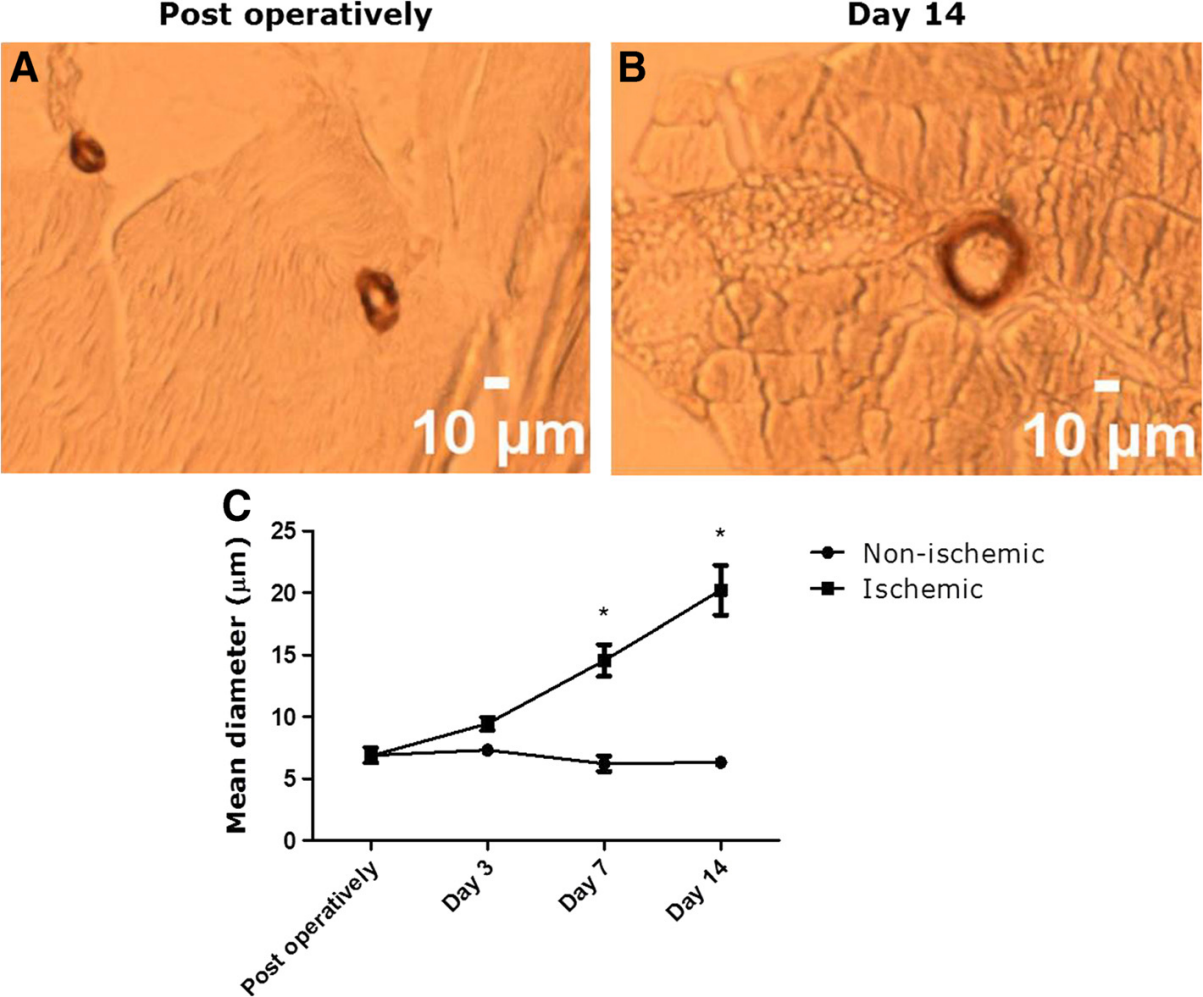
Increasing collateral diameter over time in ischemic adductor muscles. Representative pictures of collateral arteries are shown immediately after ligation (**a**) and at day 14 post ligation (**b**). Compared to the non-ischemic limb, a significant increase in collateral diameter was found at day 7 and 14 post ligation (**c**)

To evaluate post femoral artery ligation changes in collateral artery size in harvested adductor muscles, we stained for αSMA (Fig. 4a, b). We found a significant increase in mean collateral artery diameter at day 7 and day 14 post ligation while collateral artery diameter of the contralateral control limb remained stable over time (Fig. 4c).

**Fig. 5:**
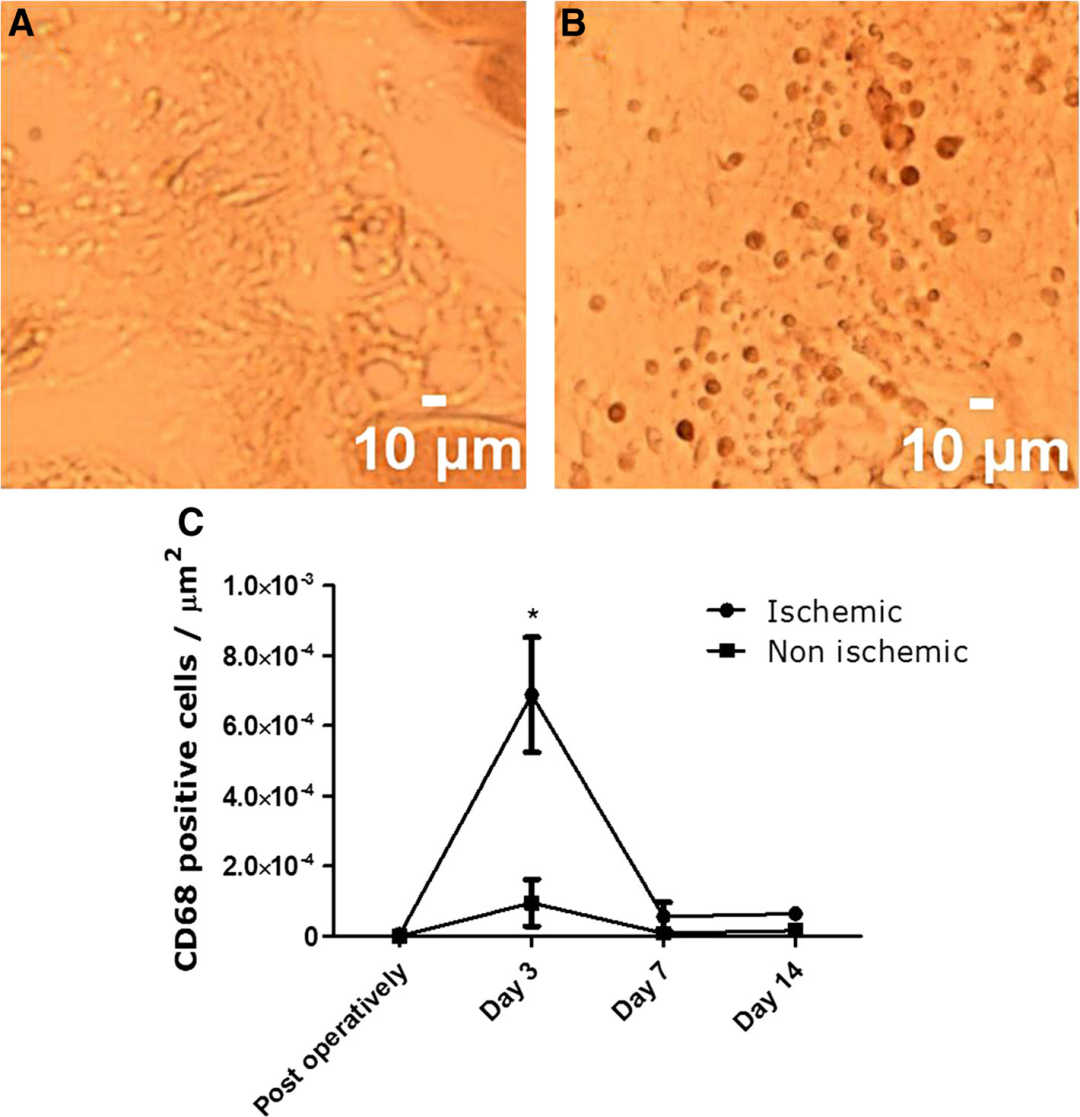
Monocyte/macrophage infiltration in the peri-muscular fascia over time. Panel **a** (non-ischemic) and **b** (ischemic) show representative images of the CD68 staining 3 days after ligation surgery. Significantly higher macrophage infiltration compared to the non-ischemic limb was found on day 3 (*p* < 0.001) (**c**). On all other days, no significant difference was found

To assess (clearance of) muscular damage in the adductor muscle of the ischemic hind limb, a CD68 monocyte/macrophage staining was performed (Fig. 5a, b). Peak macrophage infiltration in the peri-muscular fascia in the adductor muscle of the ischemic limb was observed at day 3 post ligation with numbers significantly higher compared to the non-ischemic limb. Macrophage infiltration decreased strongly at day 7 after ligation and remained decreased at day 14 after ligation. ANOVA analysis revealed no significant differences in CD68 positivity at day 7 and 14 after ligation between ischemic and non-ischemic limbs (Fig. 5c).

**Fig. 6:**
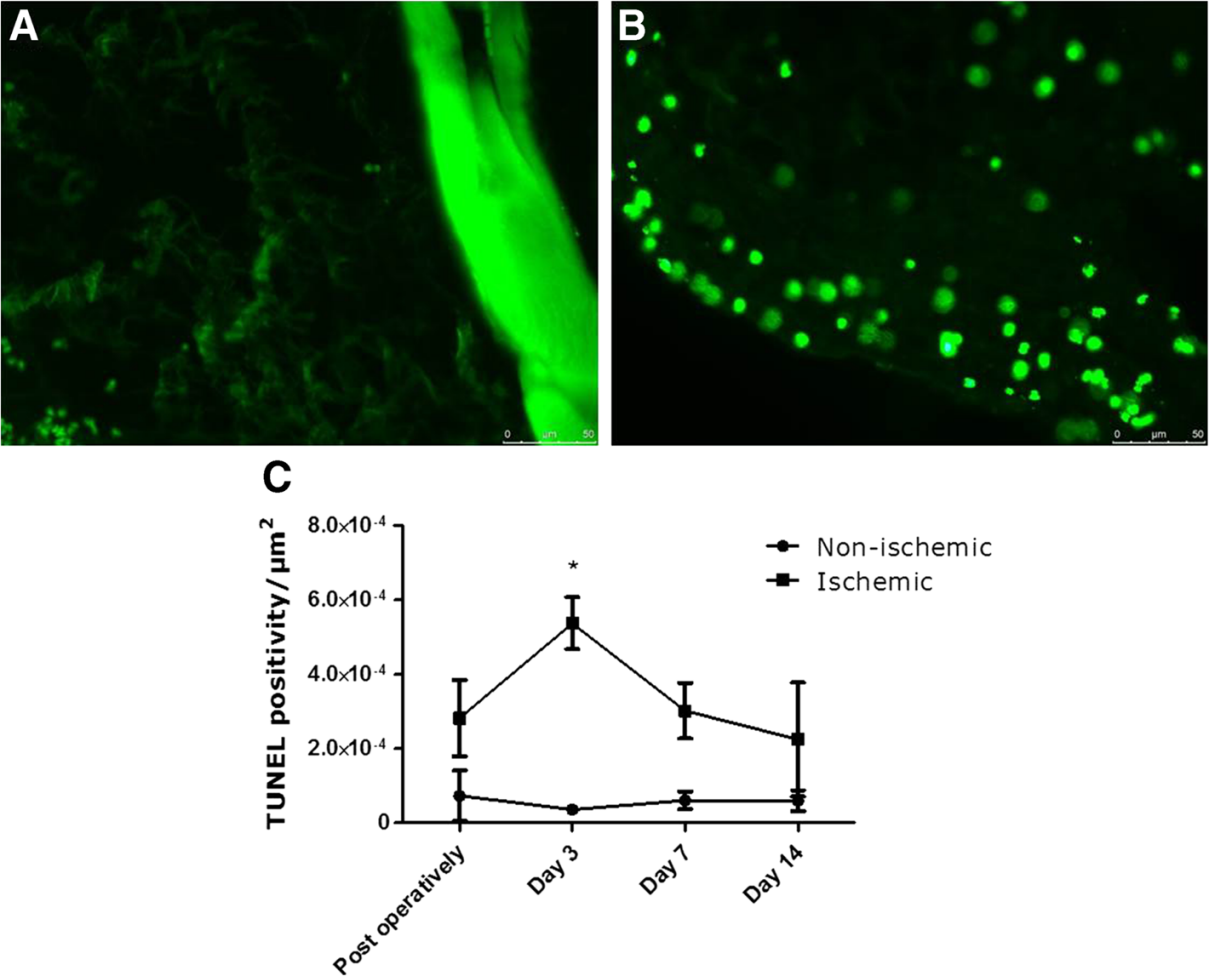
DNA fragmentation detected by TUNEL staining in ischemic and non-ischemic adductor muscles. Panel **a** (non-ischemic) and **b** (ischemic) show representative images of the TUNEL staining 3 days after ligation surgery. Significantly higher TUNEL positivity in the ischemic limb compared to the non-ischemic limb was found on day 3 after ligation (*p* < 0.001) (**c**). On all other days, no significant difference was found

To further assess muscular damage in the adductor muscle of the ischemic hind limb, we performed a TUNEL staining (Fig. 6a, b). The TUNEL positivity in the peri-muscular fascia of the ischemic limb followed a similar trend compared to the ^99m^Tc-PyP uptake. Post-operatively, TUNEL positivity was non-significantly increased in the ischemic limb compared to the control limb, while 3 days after ligation surgery, we observed a significant increase in TUNEL positivity (*p* < 0.001) in the ischemic limb. On day 7 and 14, TUNEL positivity decreased again to non-significant levels compared to the control limb (Fig. 6c).

## Discussion

This study demonstrates that perfusion recovery as assessed by ^99m^Tc-sestamibi SPECT appears much faster than LDPI data suggest. The therapeutic window in an ischemic hind limb model for the induction of arteriogenesis might therefore be drastically shorter than indicated by standard LDPI analysis.

Perfusion recovery analysis using nuclear imaging has previously been investigated in a hind limb ischemia model in rats [15] and mice [13]. While these studies used a single time point to indicate perfusion recovery, they succeeded to show that the scintigraphic enhancement of perfusion was accompanied by morphological changes (histology) and increased vascularity (angiography) indicating ongoing perfusion recovery [13, 15]. In this study, we subjected two groups of mice to LDPI and SPECT scans to assess perfusion recovery at five different time points: baseline, post-operatively, day 3, 7, and 14, thereby providing a unique insight into the temporal changes in perfusion recovery throughout the depth of the limb. Using LDPI, we found an ongoing recovery in perfusion 14 days post ligation of the femoral artery, while the ^99m^Tc-sestamibi SPECT data show nearly complete recovery of blood perfusion 7 days after ligation. Additionally, these findings were supported by postmortem histological analysis. In harvested adductor muscle tissue, we found a significantly increased collateral artery diameter at day 7 and 14 post ligation.

LDPI and SPECT measure different physiological parameters. LDPI measures blood velocity whereas ^99m^Tc-sestamibi SPECT imaging detects steady state distribution of ^99m^Tc-sestamibi and is therefore a representation of present blood volume and perfusion through uptake of the tracer by living and perfused cells. Hence, regardless of the low flow velocity, perfused collaterals will be detected by ^99m^Tc-sestamibi SPECT scans while LDPI might overlook or underestimate this perfusion. Moreover, ^99m^Tc-sestamibi SPECT imaging is able to indicate the perfusion over the entire volume of the hind limb whereas LDPI only reaches a tissue depth of a few hundred micrometers [8]. The former is obviously preferred as the majority of perfusion recovery occurs subcutaneously.

Additionally, skin and muscle perfusion are differently regulated and perhaps react differently to ligation-induced ischemia. Beta-2 (β2) adrenergic receptors residing on muscular arterioles cause vasodilation in response to sympathetic nervous-induced norepinephrine release while alpha-1 and alpha-2 adrenergic receptors on cutaneous arterioles cause vasoconstriction. A close correlation between sympathetic activation (causing norepinephrine release) and myocardial ischemia has already been shown [24, 25]. Considering the discrepancy between the LDPI and SPECT data, it is conceivable that ligation-induced ischemia triggers the sympathetic nervous system to release norepinephrine, subsequently causing cutaneous vasoconstriction and thereby creating an apparent delay in reperfusion on LDPI.

The ^99m^Tc-PyP SPECT data showed peak muscular damage 3 days post ligation and a drastic decrease in uptake 7 days after ligation. Fourteen days post ligation, ^99m^Tc-PyP SPECT data showed a nearly complete recovery. The increase in uptake of ^99m^Tc-PyP is likely to come from its binding to hydroxyapatite crystals in damaged myocytes as well as its uptake in macrophages coordinating the clearance of damaged tissue. In support of this theory and our ^99m^Tc-PyP SPECT data, we found peak macrophage infiltration in the peri-muscular fascia in the adductor muscle of the ischemic hind limb at day 3 post ligation. Macrophage numbers continued to decrease after the peak at day 3 in the ischemic hind limb thereby explaining the decrease in uptake of ^99m^Tc-PyP. The rapid clearance of muscular damage after peak levels on day 3 was further underlined by data obtained from our TUNEL staining. TUNEL positivity in the adductor muscle of the ischemic limb followed a similar trend as the ^99m^Tc-PyP uptake. We observed a significantly higher TUNEL positivity in the ischemic limb compared to the non-ischemic limb on day 3 after ligation. TUNEL positivity on day 7 and 14 after ligation were non-significantly different compared to the non-ischemic limb. Interestingly, uptake of ^99m^Tc-sestamibi roughly mirrored ^99m^Tc-PyP uptake. This indicates that the lowest level of perfusion coincides with the highest level of muscular damage. Furthermore, our data show that the (near) completion of perfusion recovery precedes clearance of damaged myocytes and subsequent muscular recovery.

So far, extremity ischemia and subsequent neovascularization-guided perfusion recovery in animal models has often been evaluated by predominantly anatomic (MRI), invasive (angiography), or limited (LDPI) techniques. Moreover, accurate documentation of tissue ischemia and perfusion recovery by means of histology requires the collection, and often the destruction of tissue samples, thereby providing limited clinical relevance and prohibiting serial monitoring of the biologic processes in living animals [26]. Nuclear perfusion and myocyte damage imaging might provide a useful and non-invasive alternative to evaluate serial changes in tissue perfusion. The feasibility and accuracy of nuclear perfusion imaging has already been demonstrated by Stacy et al. in a pig model of limb ischemia [27].

The benefit of perfusion imaging in the clinic using SPECT has already been proven. ^99m^Tc-sestamibi imaging in patients has revealed improved sensitivity for detecting differences in resting perfusion between the lower extremities of peripheral vascular disease patients with unilateral disease and improved sensitivity compared with Doppler ultrasound for the detection of peripheral vascular disease [28, 29]. Moreover, ^99m^Tc-sestamibi was also used for the evaluation of regional blood supply of the thigh and calf muscles in early stages of atherosclerosis [30, 31]. A reduced stress and rest perfusion of the lower limb muscles could be documented in clinically asymptomatic patients with atherosclerotic changes of lower limb vessels. Therefore, this technique could help early detection of the ischemic changes in asymptomatic patients, thereby enabling early intervention and prevention of disease progression.

Furthermore, ^99m^Tc-PyP has been used to estimate the ischemic skeletal muscle mass in ischemia-reperfusion injury [32]. In another study, ^99m^Tc-PyP SPECT was employed to measure the amount of regional skeletal muscle necrosis in patients [33]. It was speculated that the volume of necrosis determined by this method could predict the clinical outcome.

Our SPECT data shed a new light on studies relying on LDPI results as a read out parameter to indicate a therapeutic effect or window. The LDPI data we obtained largely resemble those reported in other studies [10, 34] and therefore point toward a similar course of perfusion recovery. However, the presented SPECT data, validated with postmortem tissue analysis, indicate recovered perfusion only 7 days post-operatively and clearance of muscular damage 14 days post-operatively.

## Conclusions

In conclusion, in this study, we showed that LDPI strongly overestimates the created therapeutic window in the ischemic hind limb model. Overestimation of the therapeutic window might lead to misinterpretation of the effect of arteriogenesis stimulating agents or application at the wrong time point as relevant perfusion recovery might have already taken place while LDPI still indicates the need for perfusion improvement. Future studies testing neovascularization stimulating agents in a hind limb model for profound ischemia should take into account that the window of opportunity is short and that this window can be ascertained using molecular perfusion/myocyte damage tracers like ^99m^Tc-sestamibi and/or ^99m^Tc-PyP.

## Abbreviations

^99M^Tc-PyP, ^99m^Tc-pyrophosphate; LDPI, laser Doppler perfusion imaging; SUV, standardized uptake value; TUNEL, terminal deoxynucleotidyl transferase dUTP nick end labeling; αSMA, α-smooth muscle actin.
